# Maternal Dietary Choices Might Impact Intrauterine Healing Processes and Postnatal Phenotype and Function in Human Fetuses with Spina Bifida Aperta—Early Clinical Observations and Implications from a Retrospective Cohort Study

**DOI:** 10.3390/biomedicines13112791

**Published:** 2025-11-16

**Authors:** Thomas Kohl

**Affiliations:** German Center for Fetal Surgery & Minimally-Invasive Therapy (DZFT), Mannheim University Hospital, Theodor-Kutzer-Ufer 1-3, 68167 Mannheim, Germany; thomas.kohl@umm.de; Tel.: +49-175-597-1213

**Keywords:** fetus, spina bifida, gastroschisis, prophylaxis, diet, vegan, vegetarian, meat, poultry, fetal surgery, fetoscopy

## Abstract

**Background:** The severity of postnatal symptoms in patients with spina bifida aperta (SBA) is also determined by secondary factors that damage the exposed neural tissue throughout gestation. The purpose of this report is to present clinical cases, from 2010 to 2025, and a new hypothesis for a nonsurgical means of prenatal secondary prophylaxis. **Patients:** Eight fetuses underwent minimally invasive fetoscopic patch closure of SBA. After delivery, an unusual degree of prenatal patch healing was observed. Furthermore, time to complete postnatal skin closure was shorter (mean ± SD: 22.00 ± 6.53 days) than in 31 contemporary patients without dietary restrictions (Mean ± SD: 44.35 ± 11.91 days; *p* < 0.001). Four of the eight prenatally operated women reported that they ate plant-based food most of the time but also some meat throughout gestation; the other four were strict vegetarians. Two other fetuses with SBA at the level of the second and third lumbar vertebrae, respectively, had not undergone prenatal surgery. Following delivery, they presented with a markedly preserved surface of the neural cord and exhibited L5 motor function. One mother of the postnatally operated patients was on a vegetarian diet; the other one on a vegan diet. **Conclusions:** These early clinical observations point to the possibility that maternal plant-based diets might ameliorate the loss of neurological function and facilitate wound healing in human fetuses with SBA. If this impact of maternal dietary habits holds true, it opens the door to a far-reaching, easily available, non-invasive secondary prophylaxis in prenatally operated and unoperated fetuses with SBA and some other malformations.

## 1. Introduction

Spina bifida aperta (SBA) is one of the most serious congenital malformations in humans; its name was coined by Nicolas Tulp [1]. Based on the lesion level, it more or less significantly impacts the postnatal quality of life of affected patients [2]. As a means of primary prevention, folic acid has been effective in order to prevent amenable SBA cases [3].

**Figure 1:**
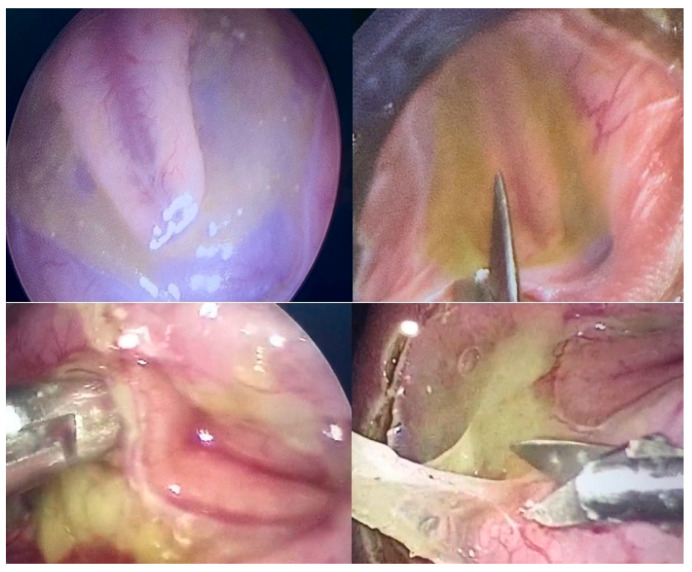
**– Supporting evidence:** The two top images highlight the preferential adherence of fetal stool (beige) to the arachnoid tissue immediately adjacent to the exposed neural cords at mid-gestation [8]. – In some other cases, like the one presented by the two bottom images, a pus-like substance engulfing the spinal cord and nerves can be observed in the subarachnoid space. The images were taken during three separate minimally invasive fetoscopic SBA surgeries performed at about 25 weeks of gestation.

Whereas the primary abnormal genetic processes that result in the development of SBA (“first hit”) occur within three weeks after conception, the postnatal severity of symptoms is significantly determined by secondary factors that damage the exposed neural tissue day by day over the course of gestation (“second hit”) [4]. Apart from mechanical damage to the neural tissue from friction, tears, and shear stress, chemical damage from changes in amniotic fluid tonicity and fetal stool has been attributed as an important “second hit” factor [5,6,7,8,9] (Figure 1).

Based on these mechanisms, various techniques for surgical closure of spina bifida during gestation were tested and developed in animal experiments before being introduced clinically [10,11,12,13,14,15,16,17]. Over the past two decades of clinical application, supported by the results of a randomized trial with one of the surgical approaches, fetal surgery for SBA has become a widely accepted means of secondary prophylaxis over the past two and a half decades, with improved neurological outcomes in many prenatally operated patients [18,19,20,21].

**Figure 2:**
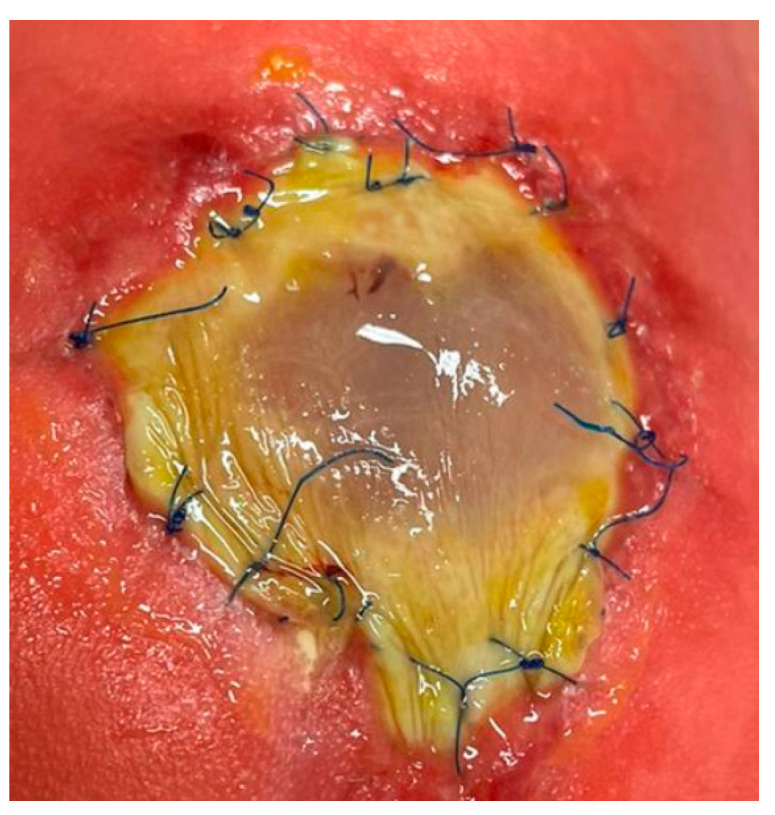
**– Supporting evidence:** Typically observed postnatal patch aspect after delivery, showing no clear healing between minimally invasive fetoscopic patch coverage of SBA and delivery, which in this case took about 10 weeks. In particular, the center of the patch looks transparent and does not exhibit a reddish hue, which would indicate the ingrowth of cells. The mother of this baby ate meat throughout gestation.

**Figure 3:**
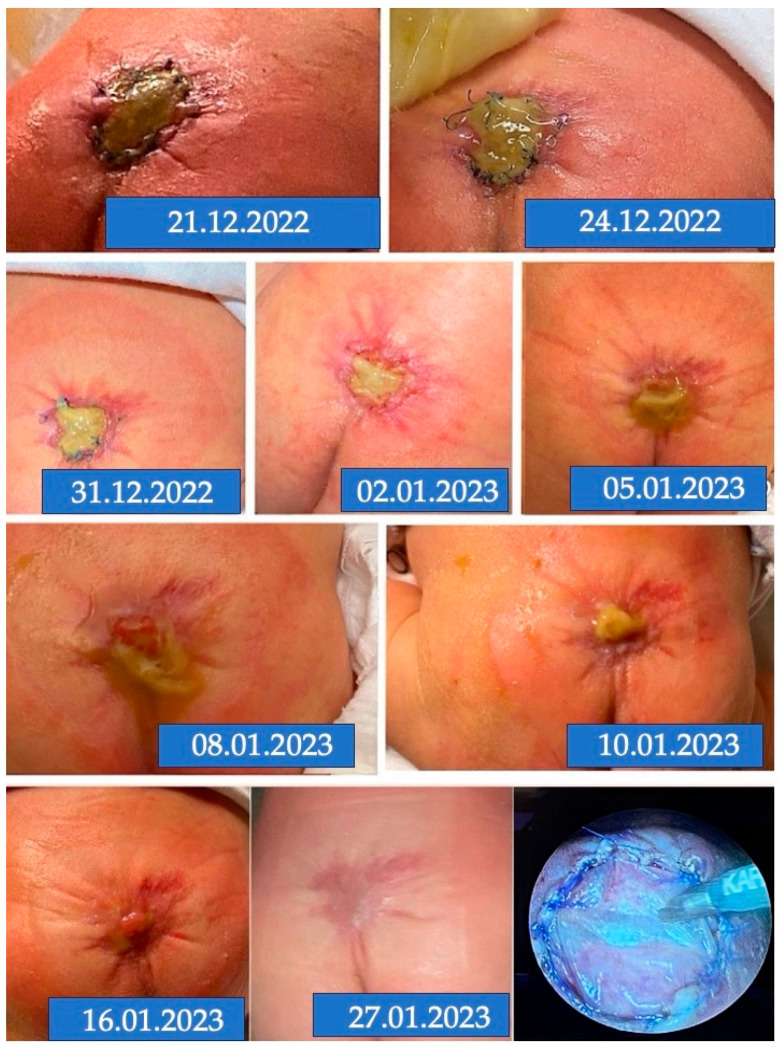
**– Control evidence:** Most commonly observed postnatal patch healing course following minimally invasive fetoscopic patch coverage of SBA and delivery beyond 33 weeks of gestation: At the time of fetal surgery at 25 + 3 weeks of gestation, the spinal cord of this L4-myelomeningocele was carefully dissected from surrounding tissues and covered water-tightly with a collagen patch (**bottom right**). The time-dated images show the healing course. After delivery, the external surface of the patch shows a thin fibrin layer but no advanced signs of healing (**top left**). Over the first two weeks of life, a quite liquid fibrinous exudate originates from the patch site. As time passes, patch healing progresses from the periphery toward the center of the patch until the patch has (almost) fully healed in (**third row right**). Taking place underneath a colloid draping, healing of the patch until complete skin closure typically takes about 6–8 weeks (**bottom middle**). Bottom right—patch at the end of prenatal surgery.

At our fetal surgery center, minimally invasive fetoscopic patch closure has been used to treat SBA during fetal life for over two decades. Based on publications of scarless fetal wound healing from the mid-nineties [22], I initially hoped that the heterograft patch, produced from porcine submucosa, that I used to cover the neural tissue would completely heal and be overgrown with skin until delivery. My optimism was fueled even more by remembering the rapid, almost complete closure of iatrogenic SBA-like lesions within several weeks after surgery that I had observed more than two decades ago during our studies in fetal sheep in order to develop the minimally invasive fetoscopic approach. Yet, to my disappointment, almost all neonates, regardless of whether they were being born weeks or even months after patch closure of their SBA, did not show signs of healing or skin overgrowth at the exterior patch surface (Figure 2). Therefore, healing of the patch until complete skin closure needed to be achieved underneath a special colloid plaster, taking about 6–8 weeks (Figure 3).

**Figure 4:**
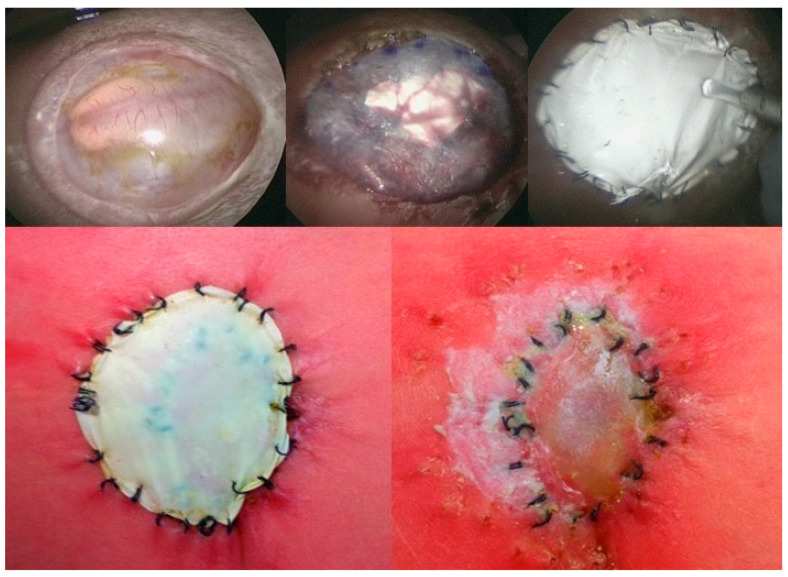
**– Supporting evidence:** Fetoscopic aspect at 27 + 2 weeks of gestation of an S1-myelomeningocele. The arachnoid adjacent to the malformed spinal cord is laced with stool (**top left**). Following resection of pathological tissue and protection of the spinal tissue with an inert Gore-Patch as well as collagen patch material (**top middle**), the entire surgical region was sealed off from the uterine milieu by an additional Gore-Patch. After delivery at 36 + 4 weeks of gestation, the upper patch is still in place (**bottom left**). Following removal of the upper patch, advanced healing at the patch site could be observed (**bottom right**). The mother stated that she had not been on a vegetarian or vegan diet and had eaten meat over the course of gestation.

In striking contrast, if, during the fetoscopic procedure, the collagen patch was covered with additional inert patch material, improved patch healing at delivery was observed, highlighting the beneficial effect on prenatal wound healing when the surgical region was protected against second-hit effects from stool and amniotic fluid (Figure 4).

## 2. Patients, Observations and Methods

Yet, over the years, every once in a while, a neonate that was born after fetoscopic surgery of SBA presented with signs of advanced healing of the patch and partial skin coverage. Unfortunately, at the time, these cases did not attract my attention as much as they should have, until a recent patient, with whom I want to start the case series of this report. As uncharted territory was explored, the description of all that follows and which resulted in arriving at a novel hypothesis that might explain a beneficial effect of maternal plant-based diets for fetuses with SBA, derives from retrospective analysis, conjecturing, and deliberate thinking, and did not follow a standardized protocol. The purpose of my manuscript is to share the observations, outcomes, and my interpretations. Yet further systematic and well-designed clinical trials will be needed to confirm the results.

### 2.1. Case 1

**Figure 5:**
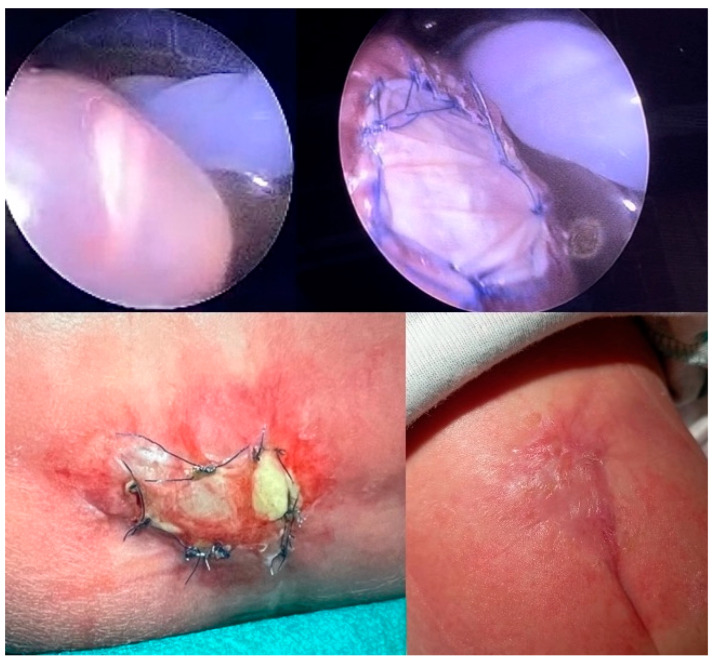
**– Case evidence 1:** Images of the case that prompted this investigation into a possible relationship between maternal dietary habits and patch healing in fetuses with SBA. At surgery at 25 + 0 weeks of gestation, the exposed neural tissue and the surrounding arachnoid tissue were free from stool adhesions (**top left**). After fetoscopic patch coverage (**top right**), the neonate was delivered at 33 + 6 weeks of gestation. On the first day of life, the patch site showed remarkably advanced healing (**bottom left**). Using our standard postnatal colloid plaster draping, complete healing was observed within thirteen days (**bottom right**), instead of the usually observed 7 weeks.

A fetus with a flat, circular myelomeningocele at the level of the fourth lumbar vertebra (L4) with a diameter of 25 mm underwent fetoscopic patch closure at 25 + 0 weeks of gestation. At surgery, the amniotic fluid was clear, and the exposed neural tissue and the surrounding arachnoid tissue were free from stool adhesions. The baby was delivered about 9 weeks after the procedure at 33 + 6 weeks of gestation because of ruptured membranes. At neurological examination, S1 motor function was observed in the neonate. Following delivery, the patch showed a remarkable degree of healing (Figure 5). Using our standardized postnatal colloid plaster draping, complete healing was observed within a record time of thirteen days.

My research for finding an explanation for this unusually fast healing process was prompted by the ingenious quantum physicist David Deutsch, who mentioned in his book “The Beginning of Infinity” [23] that there are no wonders but only a lack of explanations. His statement this time sparked my efforts to question the mother in more detail. In this interview, at one point, the mother revealed that during gestation, she had been eating a vegetarian diet “on most days of the week”. At this moment, I made the connection between her statement and the stunningly fast prenatal wound healing of large iatrogenic spina bifida-like lesions that I had observed more than two decades ago in a fetal sheep SBA model: Sheep exclusively thrive on plants.

Therefore, I looked into more detail to see whether I could find support for this hypothesis in two current cases treated at our hospital (Cases #2 and #3). Both patients were on a vegan or a vegetarian diet, respectively, and neither had undergone prenatal spina bifida surgery. From a scientific perspective, it was advantageous that both fetuses were identified to have anatomical spina bifida heights that are most commonly associated with severely impaired leg function, thus allowing a first cautious assumption about a possible association between plant-based diets and preserved function in this lesion:

### 2.2. Case 2

**Figure 6:**
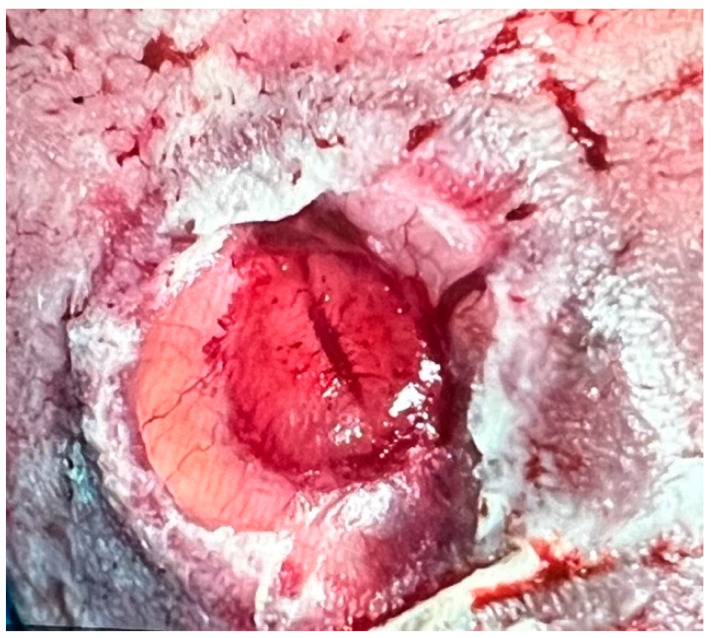
**– Case evidence 2:** Unusually well-preserved and clean neural tissue at delivery at 32 + 5 weeks of gestation in a neonate with L2-SBA whose mother had followed a strict vegan diet with vitamin supplements. The neonate underwent standard neonatal closure and exhibited L5-function.

A fetus with a flat, circular myelomeningocele at the level of the third lumbar vertebra (L2) with a diameter of 30 mm had not undergone fetal surgery, but this year was prematurely delivered at 32 + 5 weeks of gestation. At delivery, the exposed neural tissue and the surrounding arachnoid tissue were free from stool adhesions and in such remarkably preserved condition that our seasoned pediatric neurosurgeon stated that he had “never seen a cleaner malformation” than in this newborn (Figure 6). At neurological examination, L5 motor function was recorded. The mother had followed a strict vegan diet with vitamin supplements.

### 2.3. Case 3

**Figure 7:**
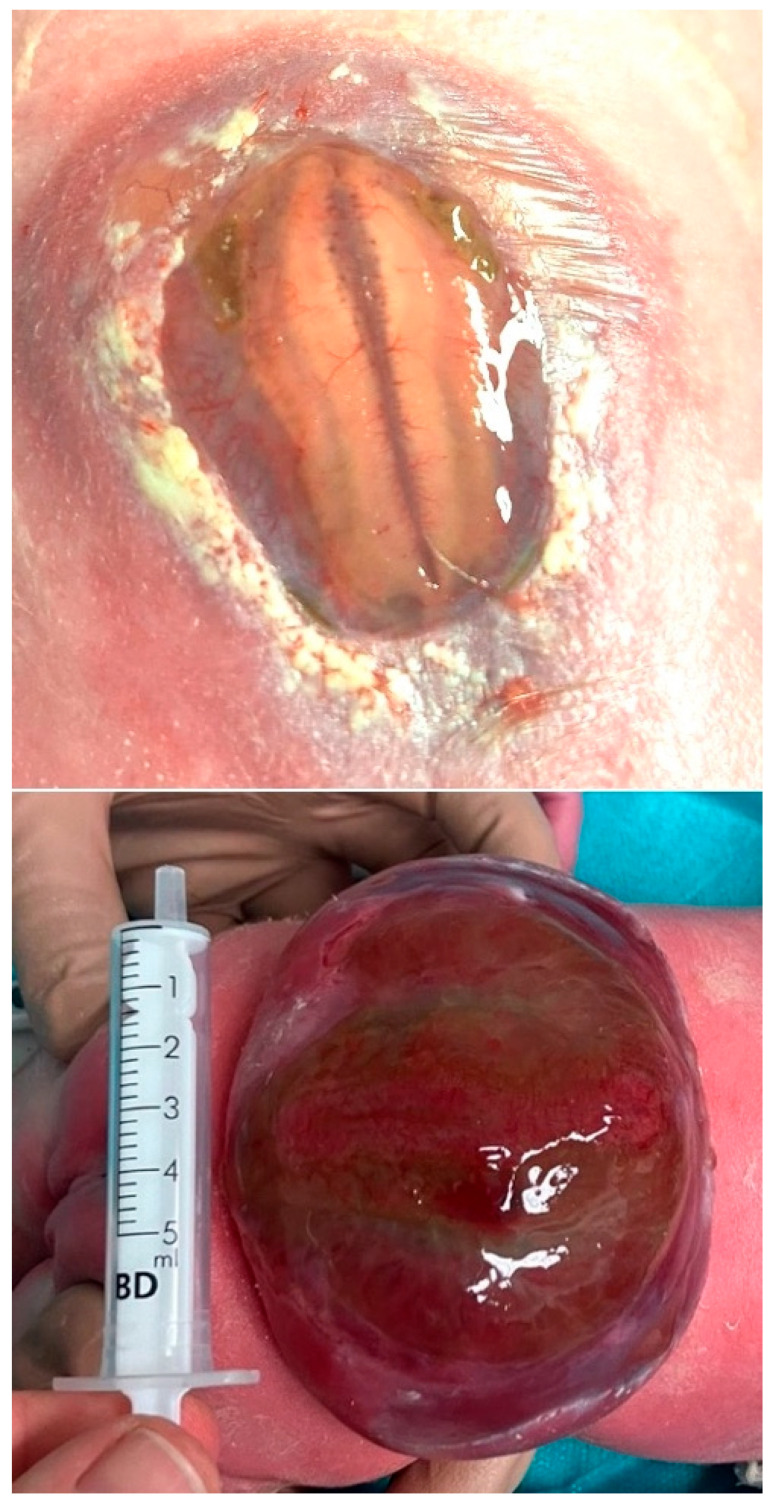
**– Case evidence 3: Top**—well-preserved but slightly stool-soiled neural tissue at delivery at 35 + 2 weeks of gestation in a neonate with L3-SBA. The mother followed a vegetarian diet. The neonate underwent standard neonatal closure and exhibited L5 function. **Bottom**—In striking contrast, destroyed, barely recognizable neural tissue was observed in another neonate with a giant L1-MMC after delivery at 34 + 1 weeks of gestation. The mother ate a diet rich in meat. In this case, paralyzed legs were already observed before 22 weeks of gestation.

A fetus with a flat, circular myelomeningocele at the level of the third lumbar vertebra (L3) with a diameter of 40 mm had not undergone fetal surgery but was prematurely delivered at 35 + 2 weeks of gestation. At delivery, the exposed neural tissue and the surrounding arachnoid tissue were slightly soiled with stool adhesions (Figure 7). At neurological examination, L5 motor function was recorded in the neonate. The mother had followed a vegetarian diet.

### 2.4. Backtesting 2010 to 2020

The records of 248 cases that were operated on between the years 2010 to 2020 were searched for images that documented improved prenatal patch healing at delivery. The following four cases were found:

### 2.5. Case 4

**Figure 8:**
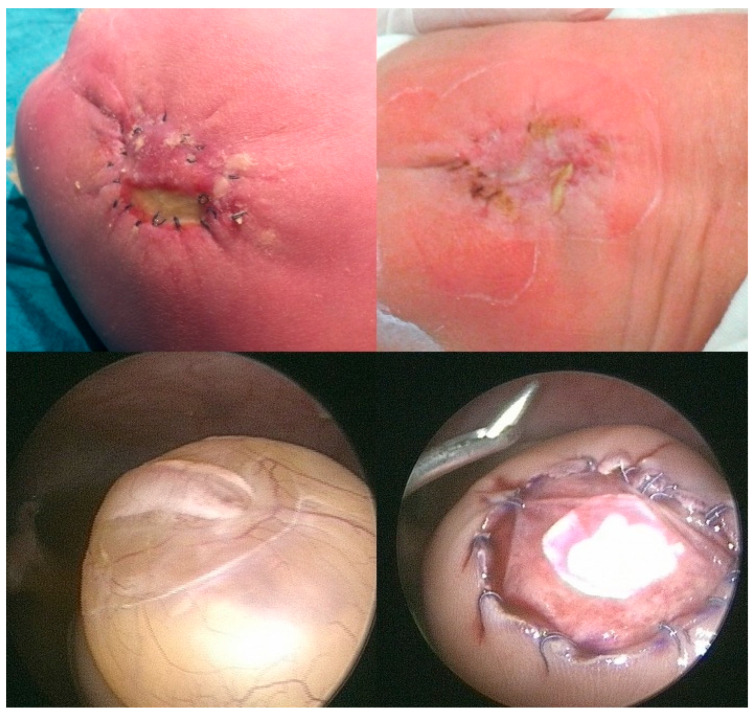
**– Case evidence 4:** Postnatal aspect of the patch area in a neonate after delivery at 38 + 3 weeks of gestation (**top left**). In this case, at surgery at 21 + 2 weeks of gestation, the exposed neural tissue and the surrounding arachnoid tissue were free from stool adhesions (**bottom left**), and fetoscopic double patch closure had been performed (**bottom right**). Using our standardized postnatal colloid plaster draping, complete healing was observed in 14 days (**top right**). The mother at the time had been mostly eating a vegetarian diet.

A fetus with a large, cystic myelomeningocele at the level of the fourth lumbar vertebra (L4) with a diameter of 25 mm underwent fetoscopic patch closure at 21 + 2 weeks of gestation. At surgery, the amniotic fluid was clear, and the exposed neural tissue and the surrounding arachnoid tissue were free from stool adhesions. The baby was electively delivered 17 weeks after the procedure at 38 + 3 weeks of gestation. Following delivery, more than half of the patch area was overgrown with skin (Figure 8). Using our standardized postnatal colloid plaster draping, complete healing was observed within 24 days. The mother remembered having “mostly eaten” a vegetarian diet.

### 2.6. Case 5

**Figure 9:**
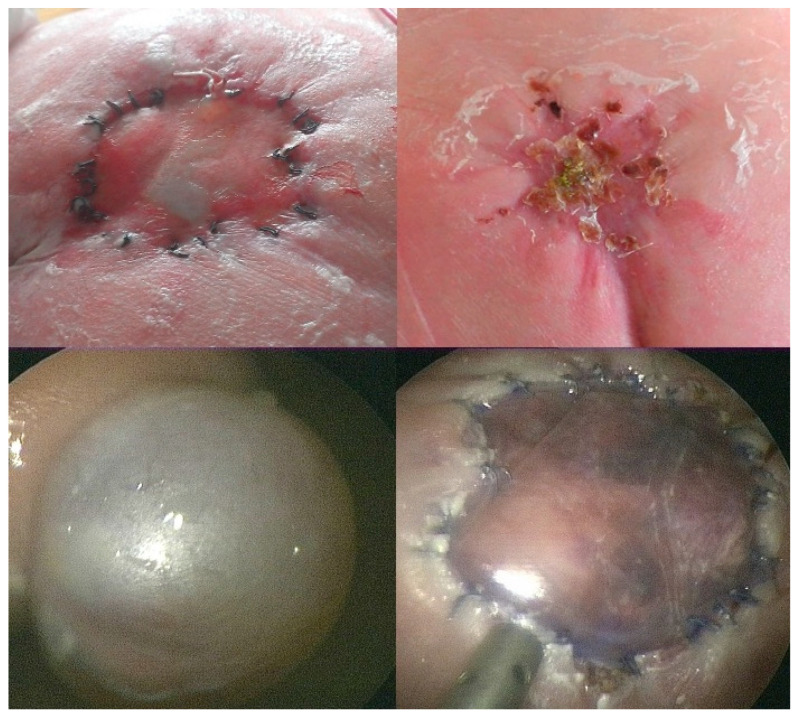
**– Case evidence 5:** Postnatal aspect of the patch area in a neonate after delivery at 35 + 4 weeks of gestation (**top left**). In this case, fetoscopic closure of a large L4-myelomeningocele had been performed at 25 + 4 weeks of gestation (**bottom left** and **right**). At surgery, the exposed neural tissue and the surrounding arachnoid tissue were free from stool adhesions. Almost complete healing was observed within 23 days (**top right**). The mother remembered eating a diet rich in plants and fibers, but also some poultry.

A fetus with a large, cystic myelomeningocele at the level of the fourth lumbar vertebra (L4) with a diameter of 40 mm underwent fetoscopic patch closure at 25 + 4 weeks of gestation. At surgery, the amniotic fluid was clear, and the exposed neural tissue and the surrounding arachnoid tissue were free from stool adhesions. The baby was delivered 10 weeks after the procedure at 35 + 4 weeks of gestation in an external hospital because of ruptured membranes. Following delivery, the patch showed a remarkable degree of healing (Figure 9). Not using our standardized postnatal colloid plaster draping, complete healing was observed within 23 days. The mother remembered eating a diet rich in plants and fibers, as well as some poultry.

### 2.7. Case 6

**Figure 10:**
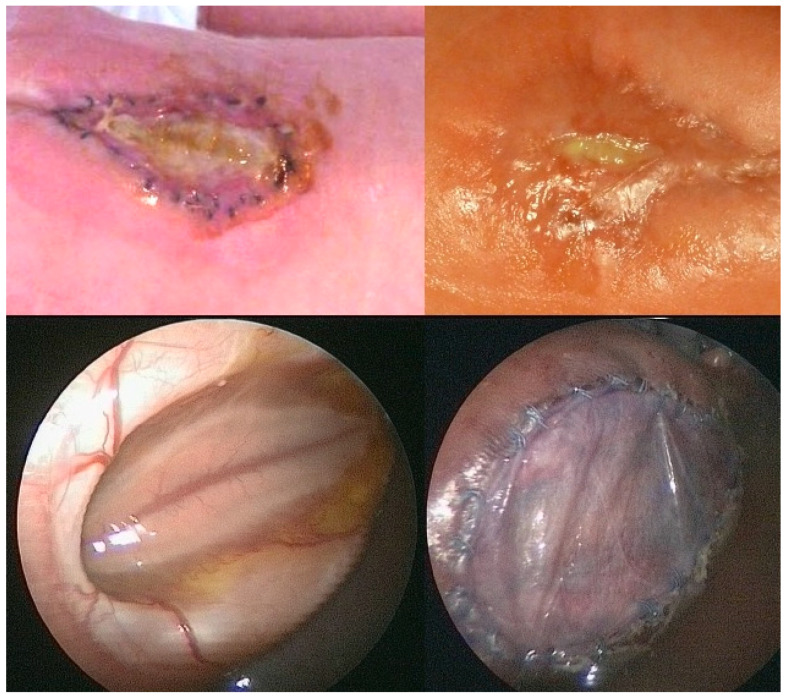
**– Case evidence 6:** Postnatal aspect of the patch area in a neonate after delivery at 33 + 2 weeks of gestation (**top left**). At the time of delivery, the rim of the patch, marked by the black surgical nitinol clips, shows already marked overgrowth toward the patch center. In this case, fetoscopic closure of a large L2 myeloschisis had been performed at 24 + 4 weeks of gestation (**bottom left** and **right**). At surgery, the exposed neural tissue and the surrounding arachnoid tissue exhibited some stool adhesions. In this case, the mother could not recollect the time it had taken the patch to completely heal (**top right**). The mother stated that she had only rarely eaten meat throughout her gestation.

A fetus with myeloschisis at the level of the second lumbar vertebra (L2) with a length of 65 mm and a width of 35 mm underwent fetoscopic patch closure at 24 + 4 weeks of gestation. At surgery, the amniotic fluid was clear, and the exposed neural tissue and the surrounding arachnoid tissue exhibited only a small amount of stool adhesions. The baby was electively delivered almost 9 weeks after the procedure at 33 + 2 weeks of gestation because of ruptured membranes. At delivery, the rim of the patch, marked by the black surgical nitinol clips, already showed marked overgrowth toward the patch center (Figure 10). The mother could not recollect how long it had taken for the patch to completely heal. Yet she remembered that she only “rarely” had eaten meat during gestation.

### 2.8. Case 7

**Figure 11:**
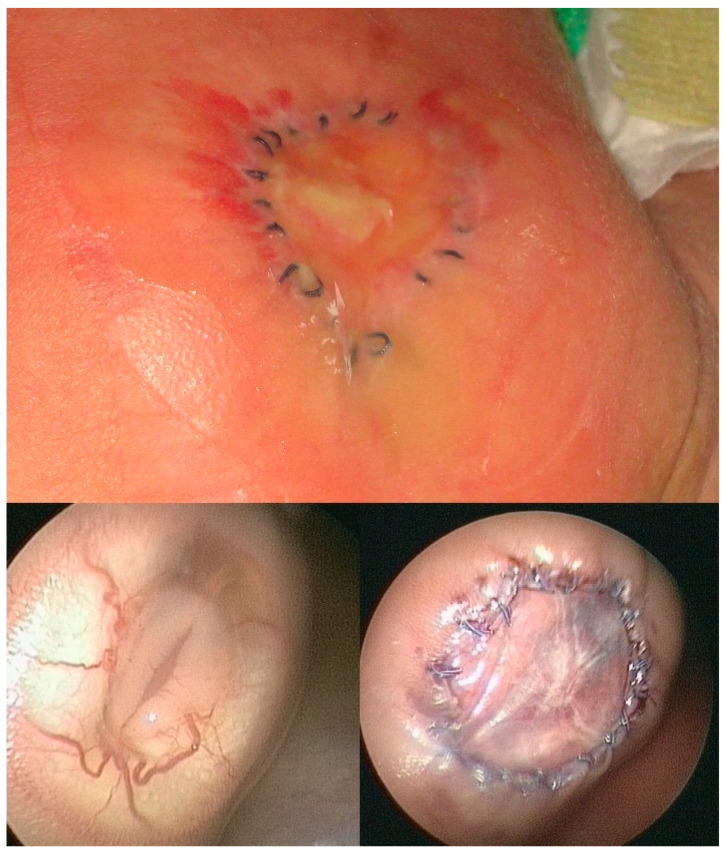
**– Case evidence 7:** Postnatal aspect of the patch area in a neonate after delivery at 31 + 6 weeks of gestation (**top**). In this case, fetoscopic closure had been performed at 23 + 0 weeks of gestation. At surgery, the exposed neural tissue and the surrounding arachnoid tissue were free from stool adhesions (**bottom left**). Using our standardized postnatal colloid plaster draping, complete healing was observed with record speed in 24 days. The mother at the time had been a vegetarian for more than a decade.

A fetus with a flat, circular myelomeningocele at the level of the first sacral vertebra (S1) with a diameter of 35 mm underwent fetoscopic patch closure at 23 + 0 weeks of gestation. At surgery, the amniotic fluid was clear, and the exposed neural tissue and the surrounding arachnoid tissue were free from stool adhesions. The baby was delivered about 8 weeks after the procedure at 31 + 6 weeks of gestation because of ruptured membranes. At neurological examination, normal motor function was observed in the neonate. Following delivery, the patch showed a remarkable degree of healing (Figure 11). Using our standardized postnatal colloid plaster draping, complete healing was observed within 24 days.

### 2.9. Backtesting 2021 to 2024

As my records from 2010 to 2020 were not complete, my overall impression remained that I had seen cases with advanced patch healing only occasionally, and as I wanted to provide a proportion that would reflect the rarity of improved patch healing, I also backtested all cases that underwent fetoscopic patch closure of SBA between January 2021 and December 2024.

A total of 46 cases were found; four cases were lost to follow-up, allowing further analysis on 91% of cases. Eight cases were excluded (two direct closure cases, one early delivery, four postnatal surgical patch revisions, and one termination of pregnancy after surgery). Of the remaining 34 cases, 3 women were vegetarians, and 31 had consumed meat during gestation.

All fetoscopic SBA surgeries were performed as standard of care at our center (DZFT). At the time of surgery, each of the expecting mothers had signed their consent to any subsequent evaluation of their case files and images for research purposes. Furthermore, the mothers of the two neonates who underwent postnatal surgery this year also gave their consent for the analysis of their files and images for this study. In order to arrive at the conclusions, no extra medical interventions were performed on any of the patients. Furthermore, no dietary recommendations had been made to any of these patients during their pregnancy.

### 2.10. Processing of Images

Apart from selecting the regions of interest from the original images, and apart from increasing brightness for better visibility and decreasing color intensity in some, no further manipulations or edits were performed on the images. The manuscript was written without the use of KI.

### 2.11. Data Management

Depending on maternal food choices during gestation, the newborns were divided into two groups. Group 1 consisted of 31 newborns who underwent fetoscopic patch closure between January 2021 and December 2024, whose mothers were not on a diet and had consumed red or processed meat during gestation. Group 2 consisted of the seven newborns that had undergone fetoscopic patch closure between January 2010 and December 2024, whose mothers had completely or mainly followed a plant-based diet during gestation, and in whom the patch healing time had been documented.

### 2.12. Statistical Analysis

For each group, the data were recorded as mean, median, standard deviation (SD), and range. The mean time in days to complete patch healing was compared between the two groups by an independent two-tailed t-test with Welch’s correction for unequal variances (primary analysis). A *p* < 0.05 was considered statistically significant.

This retrospective analysis was conducted in accordance with the Helsinki Declaration and approved on 21 August 2025 by the Ethics Committee II of the University of Heidelberg, located at Mannheim University Hospital (#2025852). The described cases were not part of any prospective research study. Furthermore, the underlying old animal studies, the pilot study for the clinical introduction of the fetoscopic surgical approach, and the retrospective analyses of all earlier outcome studies have been approved by animal research and human ethics committees.

## 3. Results

**Figure 12:**
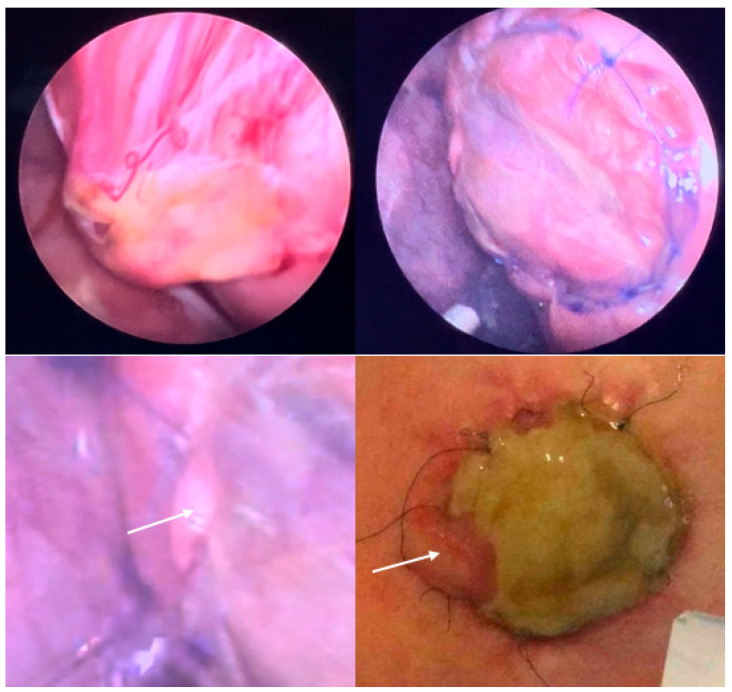
**– Control evidence:** Postnatal aspect of the patch area in a neonate after delivery at 34 + 1 weeks of gestation. In this case, fetoscopic closure of a large L2-myelomeningocele had been performed at 25 + 6 weeks of gestation (**top left** and **right**). At surgery, the exposed neural tissue was markedly altered and soiled with stool (**top left**). At the time of surgery, a small lip of skin grew into the skin, central to the suture line (white arrow—**bottom left**). At delivery, the lower left corner of the patch showed advanced healing (white arrow—**bottom right**). The healing of the entire patch took 50 days. Throughout her pregnancy, the mother had eaten meat.

All neonates of group one, with the exception of one of the 31 meat-eating mothers, did not exhibit signs of advanced patch healing after delivery, and the time between delivery and complete patch healing ranged between 26 and 68 days (mean 44 days) (Table 1). In one of these cases, following prenatal closure of a large L2-myelomeningocele at 25 + 6 weeks of gestation, at delivery at 34 + 1 weeks of gestation, the lower left rim of a patch had healed remarkably well (Figure 12). Yet, apart from that region, the patch was covered with a fibrinous layer, and complete closure of the unhealed patch part took 50 days. The mother had consumed meat during gestation. Comparing the fetoscopic image of the patch with the postnatal image taken after delivery showed that a small lip of skin had been placed over the lower left end of the patch during the fetoscopic procedure, which provided a plausible explanation for the postnatal observation of advanced healing at this location (Figure 12).

Two of the three neonates of group 2, born to the vegetarian mothers, showed advanced wound healing at delivery with complete patch healing within 22 to 31 days. In the third child of these vegetarian mothers, no postnatal image was available. In this case, the patch healing time took 27 days.

The 31 newborns of group 1 whose mothers had not followed any dietary restrictions during gestation had a significantly longer patch healing time (mean 44.35 ± 11.91 days; range 26–86 days; median 42 days) than the seven newborns of group 2 whose mothers had completely or mainly followed a plant-based diet during gestation (mean 22.00 ± 6.53 days; range 13–31 days; median 23 days; *p* = 3.32 × 10^−6^) (Table 2).

## 4. Discussion

The findings of this retrospective cohort study in human fetuses and neonates with SBA point to the possibility that maternal plant-based diets might ameliorate the loss of neurological function by lessening the chemical damage to the exposed neural tissue within the intraamniotic milieu. This connection might not only provide an explanation for why, in some prenatally untreated neonates with a fully exposed spinal cord at high lumbar levels, as in cases #2 and #3 of this series, unusually well-preserved neural tissue and leg function can be observed after delivery. To my knowledge, there have not been any explanations for these or similar observations. In contrast, similar observations are usually described as “anecdotal”, “wondrous”, “accidental”, or “incidental”, which are not explanations at all. Nevertheless, it needs to be stated right here—at the beginning of this discussion section—that spina bifida will not “heal” upon plant-based diets.

Nevertheless, the consumption of plant-based diets might also explain why better patch healing following fetoscopic patch closure of SBA was observed at delivery and why the time to complete patch healing was significantly shorter. If this relationship can be confirmed in more rigorous testing in prospective studies, it opens the door to a universally available, non-invasive means for prenatal, secondary prevention of SBA but also other malformations.

**Figure 13:**
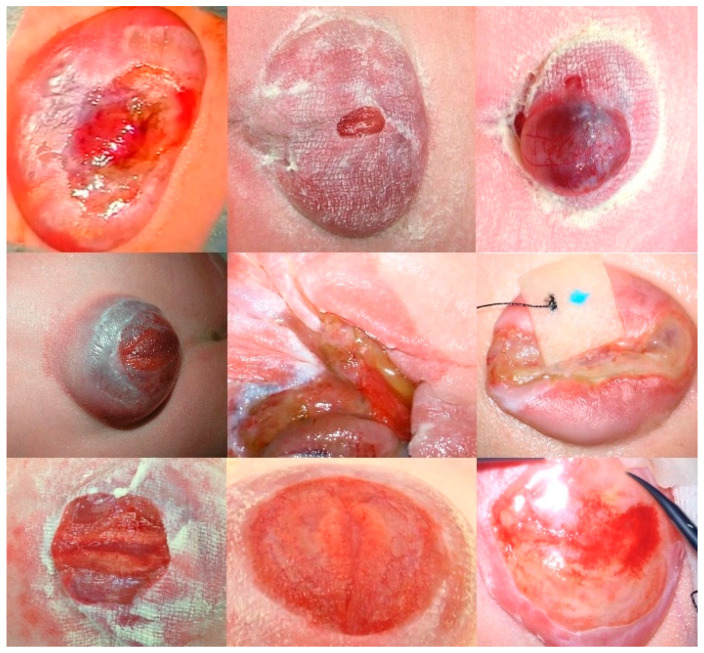
**– Supporting evidence:** Panel of the typically observed aspects of the degenerative changes to neural tissue from second-hit factors at term delivery from nine randomly selected neonates with lumbosacral myelomeningoceles. The degree of destruction stands in stark contrast to the more preserved neural tissues of those two cases (Figure 5 and Figure 6) whose mothers were on a vegan or vegetarian diet, respectively.

In comparison to the severely damaged and degenerated neural cords that are most commonly observed in prenatally unoperated newborns with SBA (Figure 13), the neural cords and meninges of the two newborns described in this manuscript, whose mothers ate plant-based diets only, looked unusually well preserved (Figure 6 and Figure 7). In both cases, the surface of the neural cord was shiny and vital. Furthermore, there were no excess capillaries, indicating inflammation.

The potential link between maternal dietary habits and the postnatal phenotype of SBA and its functional consequences is based on conjecturing a relationship between maternal foods and amniotic fluid and fetal stool toxicity [5,6,7,8,9]. This relationship may be based on the hypothesis that different foods may impact fetal pancreatic function and alter the spectrum and concentration of fetal digestive enzymes released with the fetal stool into the amniotic fluid. Alternatively, the neuroprotective effect of preferably plant-based diets may result from an increased materno-placento-fetal-amniotic fluid transfer of inflammation-reducing plant molecules, as well as by avoiding inflammation-inducing molecules from frying and grilling (red) meat to reach the intrauterine milieu by the same route.

Whereas some studies demonstrate that maternal diets influence the composition and amount of amniotic fluid [24,25,26,27,28,29], published studies in humans that assess and compare amniotic fluid pancreatic enzyme profiles of omnivores, vegetarians, or vegans are missing. In contrast, it is well known that anti-inflammatory and antioxidant compounds from plants may cross the placenta, among them short-chain fatty acids, omega-3 fatty acids, carotenoids, and sulforaphane [30,31,32,33,34]. And it has been similarly well known that multiple toxic byproducts from fried, grilled, or processed red meat are also being transferred by the placenta and reach the human fetus and amniotic fluid (e.g., polycyclic aromatic hydrocarbons, heterocyclic aromatic amines, N-nitroso compounds, advanced glycation end products, and acrylamide) [35,36,37,38,39,40,41,42,43,44].

Given the potential benefits that a lifestyle change might have for the secondary prevention of one of the most consequential malformations in humans, further basic and clinical research studies may aid in confirming or refuting any beneficial impacts of maternal diets on the phenotype and severity of SBA in their offspring.

### 4.1. Research Implications

During amniocentesis for genetic testing in normal and SBA fetuses, the mothers can be interviewed about their food preferences, and amniotic fluid might also be examined for the concentration and composition of digestive enzymes.

Mothers with SBA fetuses not undergoing prenatal surgery could be randomized into groups with vegan, vegetarian, poultry-, or red meat-allowing diets. At the time of delivery, a second fluid sample could be drawn and compared with the findings of the initial sample.

Mothers undergoing prenatal surgery could be randomized into groups with vegan, vegetarian, poultry-, or red meat-allowing diets. At the time of surgery, a second fluid sample could be drawn and compared with the findings of the initial sample.

After delivery, tissue and stool samples could be obtained from the lesion itself in prenatally unoperated newborns with SBA, or in the case of fetal surgery patients, from the patch site, and examined for cell damage and digestive enzymes.

Furthermore, the effects of various diets on fetal digestive enzyme production and activity and the effects on the expression of exocrine and endocrine pancreatic cell lines, as well as the presence and effects of inflammatory and non-inflammatory metabolic molecules, could be studied in in vivo experiments in omnivores and cell cultures regarding their impacts on experimental spina bifida and gastroschisis models. Exposing various spinal cord cells to amniotic fluid samples taken during different diets might allow us to identify characteristic beneficial or damaging factors and compositions, as well as information about which cells are the most vulnerable.

Comparison of the protective effects of plant-based diets versus stem-cell therapies on the exteriorized spinal cord in animal models.

Clinical studies could be performed in order to search for pancreatic stool enzyme differences or other factors in children born to mothers who ate vegan, vegetarian, or omnivorous diets throughout gestation.

Clinical studies could be performed in postnatal individuals from infancy to adulthood to assess if pancreatic stool enzyme composition and concentration differ depending on vegan, vegetarian, or omnivorous food intake.

As food preferences differ from country to country, addressing a potential relationship between SBA-phenotypes and diets from multiple countries might yield interesting results.

### 4.2. Clinical Implications

If maternal dietary habits indeed play a role in inflicting or ameliorating damage on the exposed spinal cord in fetuses with SBA, dietary recommendations and earlier delivery, following prenatal detection of the malformation, would be a more easily available means of secondary prophylaxis in order to ameliorate the loss of neurological function than fetal surgery.

In fetuses scheduled for fetal surgery, second-hit spinal cord damage could already be kept smaller by a plant-centered food regimen in the period between prenatal detection and intervention.

In fetuses with SBA undergoing minimally invasive fetoscopic patch closure, the time to complete patch healing could be shortened.

In prenatally operated SBA fetuses with large malformations that cover the width of their backs, lateral skin incisions might heal better.

As stool particles and amniotic fluid can be sucked into the CSF space via the central canal of the spinal cord, it may be possible that they are involved in associated cerebral pathologies like neuroepithelial/ependymal denudation, stenosis of cerebrospinal fluid pathways, and subcortical heterotopias [45]. It should be tested whether, preferably, plant-based diets might ameliorate these effects.

Furthermore, a preferably plant-based diet might ameliorate the development of the so-called “gastric peel” gastroschisis, from fetal stool within the amniotic cavity, which has been implicated as an important factor of damage to the eviscerated bowel loops [46,47,48].

### 4.3. Limitations

As uncharted territory was explored, arriving at a novel hypothesis that might explain a beneficial effect of maternal plant-based diets for fetuses with SBA derived from retrospective analysis, conjecturing, and deliberate thinking, and did and could not follow a standardized protocol. Therefore, arriving at definite conclusions about the impact of maternal dietary habits on postnatal malformation phenotype in fetuses with SBA following the observation and retrospective evaluation of only a few cases is still a long shot, and further systematic and well-designed clinical trials will be needed to confirm the results.

Yet, this manuscript offers a new hypothesis on why, in some newborns with SBA, a far better leg function was observed after delivery than what would have been expected from the anatomical lesion level. Furthermore, different dietary choices might offer an explanation of why only those surgical patches in the operated fetuses whose mothers entirely or mostly adhered to a plant-based diet exhibited an advanced degree of patch healing at delivery.

That it took me more than two decades to arrive at this hypothesis results from the rarity of these observations, which can also be explained by the overall low rates of pregnant women being vegetarians (6%) or vegans (2%) [49,50]. In Germany, in one study, about 6% of non-pregnant women were vegetarians [51]. And in the light of this paper’s hypothesis, it seems ironic that pregnant women, among them most certainly many with SBA fetuses, are usually encouraged to increase their meat intake during gestation by their local gynecologists because of their physiologically low hemoglobin readings and their increased demand for iron.

For the mothers of the fetuses described in this report, their vegetarian and vegan diets were lifestyle choices such that any effects attributed to these diets might also be dependent on the exposure of affected fetuses to SBA throughout their entire course of gestation. It might be possible that beginning a diet later in gestation, i.e., only following detection of the lesion beyond 20 weeks of gestation, might result in smaller degrees of clinically advantageous effects on spinal cord integrity and function, or, in the case of prenatally operated fetuses, on patch or wound healing.

The conclusions of this manuscript were drawn from self-reported, retrospective descriptions of the food choices of previous patients, some of them interviewed more than a decade following their gestations. As a result, there might be errors in remembering exactly what had been eaten at the time. Furthermore, the self-reported descriptions *vegan, vegetarian,* or *meat eaters* were not further examined by requesting detailed lists of food post hoc. The numbers were too small to allow any meaningful differentiation between different types of vegetarians (e.g., lacto-, ovo-lacto-, pesco-). Yet during the interviews, the eight previous patients that were grouped into the plant-based diet group recalled having followed that regimen all or most of the time, whereas the other ones, grouped in the meat-eating group, did not recall any meat restrictions throughout their gestations.

Nevertheless, several patients who were grouped into the preferably plant-based group admitted that they occasionally had eaten some meat. One patient remembered more specifically that she had mostly been eating a vegetarian diet but also had consumed poultry several times a week. It might be possible and, from what is known, plausible that maternal consumption of poultry may have a less toxic effect on amniotic fluid composition. In contrast to red meat, poultry is not associated with cancer, as it contains less hemoglobin than red meat and does not trigger N-nitroso-compound production in the body [52].

### 4.4. Assessment and Reduction in Risks

Further clinical observations are needed in order to confirm or refute the hypothesis that plant-based diets might have positive effects on fetal wound healing or the protection of neural tissue against damaging chemical factors within the amniotic milieu. As there are many different plant-based diets, further studies are needed to define if and which diet, a vegan or a vegetarian one, or one that would allow poultry, would achieve the most effective secondary prophylaxis. Furthermore, any dietary recommendation should be followed by follow-up studies to become aware of any detrimental or other beneficial effects that the recommendation of a specific diet during the period of human prenatal development might have on future postnatal health and pancreatic development [53].

Whereas many positive effects of plant-based diets have been found in studies in postnatal individuals, there is only limited evidence regarding the risks and benefits of these diets during gestation [54]. In one retrospective, web-based, self-reported study, maternal plant-based diets during gestation were associated with a higher risk for being too small for gestational age and a lower birth weight centile [55]. Palma and coauthors in their review mention maternal advantages of decreased rates of maternal gestational diabetes and hypertension, as well as early evidence of positive cognitive effects on their offspring [56].

In a recent review, Sebastiani and coauthors state that “the available evidence shows that well-planned vegetarian and vegan diets may be considered safe during pregnancy and lactation, but they require a strong awareness for a balanced intake of key nutrients”. The authors, therefore, recommend a strong awareness to provide sufficient amounts of iron, vitamin D, calcium, iodine, omega-3, and vitamin B12 supplements [57].

As proxies for preserved leg function, most fetuses with SBA exhibit normal-looking leg movements, normal foot position, and hypoechoic thigh and calf musculature well beyond mid-gestation. Therefore, in order to reduce any potential risks from vegan or vegetarian diets, their length could be shortened to 15 weeks between the 20th and the 35th week of gestation. This is usually the period where deterioration of leg function and the development of club feet are observed. At 36 weeks, fetuses with prenatally unoperated SBA could then be delivered. By this strategy, the prenatal period of other 2nd hit factors would also be shortened, with a very low risk for acquiring chronic sequelae from immaturity.

## 5. Conclusions

These early clinical observations point to the possibility that a maternal vegetarian or vegan diet might positively impact fetal healing processes and postnatal phenotype and neurological function in human fetuses with SBA. If this hypothesis holds true, it opens the door to a noninvasive secondary prophylaxis in prenatally operated and unoperated fetuses with SBA, as well as it may be for those with some other malformations, the first one coming to my mind being gastroschisis. Therefore, rigorous scientific studies are desired to learn more about the potential benefits and risks of extended periods of plant-based maternal foods during the critical period of human development.

## Figures and Tables

**Table 1:** **Characteristics of the 8 cases with fetoscopic surgery and the 2 cases with postnatal surgery for spina bifida aperta (SBA) with expectant mothers on plant-based diets**.

Cases Fetoscopic Surgery	Gestational Age at Fetal Surgery (Weeks + Days)	Gestational Age at Delivery (Weeks + Days)	Time to Complete Healing (Days)	Aspect of Patch at Delivery	Maternal Diet	Figure
1	25 + 0	33 + 6	13	Remarkably advanced healing	Mostly plant-based	Figure 5
2	21 + 2	38 + 3	14	Remarkably advanced healing	Mostly vegetarian	Figure 8
3	25 + 4	35 + 4	23	Remarkably advanced healing	Mostly vegetarian diet, some poultry	Figure 9
4	25 + 4	33 + 2	not recollected	Marked overgrowth	Mostly vegetarian, rarely meat	Figure 10
5	23 + 0	31 + 6	24	Marked overgrowth	Vegetarian	Figure 11
6	25 + 1	29 + 5	22	Marked overgrowth	Vegetarian	-
7	25 + 5	32 + 1	31	Marked overgrowth	Vegetarian	-
8	26 + 1	34 + 5	27	Marked centripetal overgrowth	Vegetarian	-
**Postnatal surgery**			**Level**	**Aspect of lesion** **at delivery**	**Maternal diet**	**Figure**
No fetal surgery. Standard neonatal closure	–	32 + 5	Anatomical level L2 Functional level L5	Unusually well preserved and clean neural tissue	Strictly vegan diet with vitamin supplements.	Figure 6
No fetal surgery. Standard neonatal closure.	–	35 + 2	Anatomical level L3 Functional level L5	Well preserved but slightly stool-soiled neural tissue	Vegetarian	Figure 7

**Table 2:** **Patch healing time in two cohorts of newborns after fetoscopic patch closure for spina bifida and maternal food choices during gestation**.

Group I—Patch healing in days in 31 newborns/no maternal dietary restrictions
26, 28, 30, 31, 32, 33, 36, 36, 38, 39, 40, 41, 41, 42, 42, 42, 43, 45, 46, 46, 49, 49, 50, 50, 50, 52, 56, 56, 57, 63, 86
Mean 44.35 ± 11.91 days (Range 26–86 days/Median 42 days)
Group II—Patch healing in days in 7 newborns/mainly plant-based maternal diets
13, 14, 22, 23, 24, 27, 31
Mean 22.00 ± 6.53 days (Range 13–31 days/Median 23 days)
(*p* < 0.001 in two-tailed Welch’s *t*-test)

## Data Availability

The raw data supporting the conclusions of this article will be made available to any reviewer of this manuscript on request.

## References

[1] Tulpi N. (1641). Observationum Medicarum Libri Tres. Capitum 30 Spina Dorsi Bifida.

[2] Özek M.M., Cinalli G., Maixner W.J., Özek M.M., Erol B., Tama I.J. (2008). Spina bifida Management and Outcome. Management of Vertebral Problems and Deformities.

[3] Wagh K., Kancheria V., Dorsey A., Pachón H., Oakley G.P. (2024). A global update on the status of prevention of folic-acid-preventable spina bifida and anencephaly in year 2022. Birth Defects Res..

[4] Heffez D.S., Aryanpur J., Hutchins G.M., Freeman J.M. (1990). The paralysis associated with myelomeningocele: Clinical and experimental data implicating a preventable spinal cord injury. Neurosurgery.

[5] Correia-Pinto J., Reis J.L., Hutchins G.M., Baptista M.J., Estevão-Costa J., Flake A.W., Leite-Moreira A.F. (2002). In utero meconium exposure increases spinal cord necrosis in a rat model of myelomeningocele. J. Pediatr. Surg..

[6] Drewek M.J., Bruner J.P., Whetsell W.O., Tulipan N. (1997). Quantitative analysis of the toxicity of human amniotic fluid to cultured rat spinal cord. Pediatr. Neurosurg..

[7] Danzer E., Zhang L., Radu A., Bebbington M.W., Liechty K.W., Adzick N.S., Flake A.W. (2011). Amniotic fluid levels of glial fibrillary acidic protein in fetal rats with retinoic acid induced myelomeningocele: A potential marker for spinal cord injury. Am. J. Obs. Gynecol..

[8] Kohl T. (2010). Stool contamination. J. Neurosurg. Pediatr..

[9] Athiel Y., Jouannic J.M., Lépine M., Maillet C., de Saint Denis T., Larghero J., Guilbaud L. (2024). Role of Amniotic Fluid Toxicity in the Pathophysiology of Myelomeningocele: A Narrative Literature Review. Prenat. Diagn..

[10] Selauki M., Manning S., Bernfield M. (2001). The curly tail mouse model of human neural tube defects demonstrates normal spinal cord differentiation at the level of the myelomeningocele: Implications for fetal surgery. Child’s Nerv. Syst..

[11] Michejda M. (1984). Intrauterine treatment of spina bifida: Primate model. Eur. J. Pediatr. Surg..

[12] Michejda M. (1989). Antenatal treatment of central nervous system defects: Current and future developments in experimental therapies. Fetal Ther..

[13] Meuli M., Meuli-Simmen C., Yingling C.D., Hutchins G.M., Hoffman K.M., Harrison M.R., Adzick N.S. (1995). Creation of myelomeningocele in utero: A model of functional damage from spinal cord exposure in fetal sheep. J. Pediatr. Surg..

[14] Meuli M., Meuli-Simmen C., Yingling C.D., Hutchins G.M., Timmel G.B., Harrison M.R., Adzick N.S. (1996). In utero repair of experimental myelomeningocele saves neurological function at birth. J. Pediatr. Surg..

[15] Kohl T., Witteler R., Strümper D., Gogarten W., Asfour B., Reckers J., Merschhoff G., Marcus A.E., Weyand M., Van Aken H. (2000). Operative techniques and strategies for minimally invasive fetoscopic fetal cardiac interventions in sheep. Surg. Endosc..

[16] Kohl T., Große Hartlage M.G., Kienitz D., Westphal M., Buller T., Achenbach S., Aryee S., Gembruch U., Brentrup A. (2003). Percutaneous fetoscopic patch coverage of experimental lumbosacral full- thickness skin lesions in sheep—A minimally invasive technique aimed at minimizing maternal trauma from fetal surgery for myelomeningocele. Surg. Endosc..

[17] Herrera S.R., de Almeida Leme R.J., Valente P.R., Caldini E.G., Saldiva P.H., Lapa Pedreira D.A. (2012). Comparison between two surgical techniques for prenatal correction of meningomyelocele in sheep. Einstein.

[18] Adzick N.S., Thom E.A., Spong C.Y., Brock J.W., Burrows P.K., Johnson M.P., Howell L.J., Farrell J.A., Dabrowiak M.E., Sutton L.N. (2011). A randomized trial of prenatal versus postnatal repair of myelomeningocele. N. Engl. J. Med..

[19] Kohl T. (2014). Percutaneous minimally invasive fetoscopic surgery for spina bifida aperta. Part I: Surgical technique and perioperative outcome. Ultrasound Obs. Gynecol..

[20] Diehl D., Belke F., Kohl T., Axt-Fliedner R., Degenhardt J., Khaleeva A., Oehmke F., Faas D., Ehrhardt H., Kolodziej M. (2021). Fully Percutaneous Fetoscopic Repair of Myelomeningocele: 30 months follow up data. J. Ultrasound Obs. Gynecol..

[21] Cortes M.S., Chmait R.H., Lapa D.A., Belfort M.A., Carreras E., Miller J.L., Brawura Biskupski Samaha R., Gonzalez G.S., Gielchinsky Y., Yamamoto M. (2021). *Experience of* 300 cases of prenatal fetoscopic open spina bifida repair: Report of the International Fetoscopic Neural Tube Defect Repair Consortium. Am. J. Obs. Gynecol..

[22] Lorenz H.P., Adzick N.S. (1993). Scarless skin wound repair in the fetus. West. J. Med..

[23] Deutsch D. (2011). The Beginning of Infinity. Explanations that Transform the World.

[24] Spill M., Callahan E., Johns K., Shapiro M., Spahn J.M., Wong Y.P., Terry N., Benjamin-Neelon S., Birch L., Black M. (2019). Influence of Maternal Diet on Flavor Transfer to Amniotic Fluid and Breast Milk and Children’s Responses: A Systematic Review. Am. J. Clin. Nutr..

[25] Niu X., Lu D., Jaleel S., Palmer S.N., Mahendroo M., Zhan X., Mirpuri J. (2024). Maternal high fat diet exposure modifies amniotic fluid metabolites and expands 3 innate lymphoid cells dependent on the maternal microbiome and MyD88-signaling. Front. Immunol..

[26] Ventura A.K., Worobey J. (2013). Early influences on the development of food preferences. Curr. Biol..

[27] Cheung C.Y., Roberts V.H.J., Frias A.E., Brace R.A. (2018). High-fat die effects on amniotic fluid volume and amnion aquaporin expression in non-human primates. Physiol. Rep..

[28] Priyadarhini M., Thomas A., Reisetter A.C., Wolever M.S., Josefson J.L., Layden B.T. (2014). Maternal short-chain fatty acids are associated with metabolic parameters in mothers and newborns. Transl. Res..

[29] Larqué E., Demmelmair H., Gil-Sánchez A., Prieto-Sánchez M.T., Blanco J.E., Pagán A., Faber F.L., Zamora S., Parrilla J.J., Koletzko B. (2011). Placental transfer of fatty acids and fetal implications. Am. J. Clin. Nutr..

[30] Middleton P., Gomersall J.C., Gould J.F., Shepherd E., Olsen S.F., Makrides M. (2018). Omega-3 fatty acid supplementation during pregnancy. Cochrane Database Syst. Rev..

[31] Zielińska M.A., Wesolowska A., Pawlus B., Harnulka J. (2017). Health Effects of Carotenoids during Pregnancy and Lactation. Nutrients.

[32] de Souza Mesquita L.M., Mennitti L.V., de Rosso V.V., Pisani L.P. (2021). The role of vitamin A and its pro-vitamin carotenoids in fetal and neonatal programming: Gaps in knowledge and metabolic pathways. Nutr. Rev..

[33] Black A.M., Armstrong E.A., Scott O., Juurlink B.J.H., Yager J.Y. (2015). Broccoli sprout supplementation during pregnancy prevents brain injury in the newborn rat following placental insufficiency. Behav. Brain Res..

[34] Ladak Z., Garcia E., Yoon J., Landy T., Armstrong E.A., Yager J.Y., Persad S. (2021). Sulforaphane (SFA) protects neuronal cells from oxygen & glucose deprivation (OGD). PLoS ONE.

[35] Li Z., Han Y., Li X., Xiang W., Cui T., Xi W., Jin S., Zhan X. (2025). Polycyclic aromatic hydrocarbons in early pregnancy on child neurodevelopment. Environ. Pollut..

[36] Syeda T., Cannon J.R. (2022). Potential Role of Heterocyclic Aromatic Amines in Neurodegeneration. Chem. Res. Toxicol..

[37] Turesky R.J. (2002). Heterocyclic aromatic amines: Metabolism, DNA adducts, formation, mutagenesis, and carcinogenesis. Drug Metab. Rev..

[38] Liu J., Xu W., Liu Y., Zhang Q. (2025). Reproductive and developmental toxicology of nitrosamines. Toxicol. Res..

[39] Huncharek M., Kupelnick B. (2004). A meta-analysis of maternal cured meat consumption during pregnancy and the risk of childhood brain tumors. Neuroepidemiology.

[40] von Stedingk H., Vikström A.C., Rydberg P., Pedersen M., Nielsen J.K., Segerbäck D., Knudsen L.E., Törnqvist M. (2011). Analysis of hemoglobin adducts from acrylamide, glycidamide, and ethylene oxide in paired mother/cord blood samples from Denmark. Chem. Res. Toxicol..

[41] Erdemli M.E., Aladag M.A., Altinoz E., Demirtas S., Turkoz Y., Yigitcan B., Bag H.G. (2018). Acrylamide applied during pregnancy causes the neurotoxic effect by lowering BDNF levels in the fetal brain. Neurotoxicol. Teratol..

[42] Zhao M., Zhang B., Deng L. (2022). The Mechanism of Acrylamide-Induced Neurotoxicity: Current Status and Future Perspectives. Front. Nutr..

[43] Uribarri J., Woodruff S., Goodman S., Cai W., Chen W., Chen X., Pyzik R., Yong A., Striker G.E., Vlassara H. (2010). Advanced glycation end products in foods and a practical guide to their reduction in the diet. J. Am. Diet. Assoc..

[44] Vincent A.M., Perrone L., Sullivan K.A., Backus C., Sastry A.M., Lastoskie C., Feldman E.L. (2007). Receptor for Advanced Glycation End Products Activation Injures Primary Sensory Neurons via Oxidative Stress. Endocrinology.

[45] Sival D.A., Montserrat G., den Dunnen W.F.A., Bátiz L.F., Alvial G., Castañeyra-Perdomo A., Rodríguez E.M. (2011). Neuroependymal denudation is in progress in full-term human foetal spina bifida aperta. Brain Pathol..

[46] Correia-Pinto J., Tavares M.L., Baptista M.J., Henriques-Coelho T., Estevão-Costa J., Flake A.W., Leite-Moreira A.F. (2002). Meconium dependence of bowel damage in gastroschisis. J. Pediatr. Surg..

[47] Olguner M., Akgür F.M., Api A., Ozer E., Aktug T. (2000). The effects of intraamniotic human neonatal urine and meconium on the intestines of the chick embryo with gastroschisis. J. Pediatr. Surg..

[48] Luton D., De Lagausie P., Guibourdenche J., Oury J.F., Vuillard E., Sibony O., Farnoux C., Aigrain Y., Blot P. (1997). Prognostic factors of prenatally diagnosed gastroschisis. Fetal Diagn. Ther..

[49] Yisahak S.F., Hinkle S.N., Mumford S.L., Li M., Andriessen V.C., Grantz K.L., Zhang C., Grewal J. (2020). Vegetarian diets during pregnancy, and maternal and neonatal outcomes. Int. J. Epidemiol..

[50] Przybysz P., Kruszewski A., Kacperczyk-Barnik J., Romejko-Wolniewicz E. (2023). The Impact of Maternal Plant-Based Diet on Obstetric and Neonatal Outcomes—A Cross-Sectional Study. Nutrients.

[51] Mensink G.B.M., Barbosa C.L., Brettschneider A.K. (2016). Prevalence of persons following a vegetarian diet in Germany. J. Health Monit..

[52] Food Safety Authority of Ireland (2015). IARC Report: Red Meat, Processed Meat and Cancer.

[53] Hill D.J., Hill D.G. (2024). Maternal diet during pregnancy and adaptive changes in the maternal and fetal pancreas have implications for future metabolic health. Front. Endocrinol..

[54] Meulenbroeks D., Otten E., Smeets S., Groeneveld L., Jonkers D., Eussen S., Scheepers H., Gubbels J. (2024). The Association of a Vegan Diet during Pregnancy with Maternal and Child Outcomes: A Systematic Review. Nutrients.

[55] Kesary Y., Avital K., Hiersch L. (2020). Maternal plant-based diet during gestation and pregnancy outcomes. Arch. Gynecol. Obs..

[56] Palma O., Jallah J.K., Mahakalkar M.G., Mendhe D.M. (2023). The Effects of Vegan Diet on Fetus and Maternal Health: A Review. Cureus.

[57] Sebastiani G., Barbero A.H., Borrás-Novell C., Casanova M.A., Aldecoa-Bilbao V., Andreu-Fernández V., Tutusaus M.P., Martínez S.F., Gómez Roig M.D., García-Algar O. (2019). The Effects of Vegetarian and Vegan Diet during Pregnancy on the Health of Mothers and Offspring. Nutrients.

